# Second branchial cleft cyst with snoring during sleep as initial symptom: A case report and literature review

**DOI:** 10.1097/MD.0000000000027037

**Published:** 2021-08-27

**Authors:** Zhixiong Xian, Yongchao Chen, Yishu Teng, Saihong Han, Lan Li

**Affiliations:** Department of Otolaryngology, Shenzhen Children's Hospital, Shenzhen, Guangdong, China.

**Keywords:** pharyngeal neoplasm, second branchial cleft cyst, sleep snoring

## Abstract

The second branchial cleft cyst lacks typical symptoms, and its clinical manifestations are complex and varied. Among them, the second branchial cleft cyst manifested by sleep snoring is relatively rare, and it can easily lead to missed diagnosis or misdiagnosis. This paper reports a case of a second branchial cleft abscess with snoring as the main manifestation. The branchial cleft cyst was removed using an endoscopic branchial cleft.

## 1 Introduction

Branchial cleft cyst (BCC) is the second most common cause of congenital cervical mass in children, followed by thyroglossal duct cysts.^[1,2]^ Depending on the site of onset, it can be classified into first, second, third, and fourth BCC. Among them, the second BCC was the most common. Studies have shown that the second BCC accounts for 95% of all BCCs.^[3,4]^ The second BCC can occur in any part from the anterior one-third of the sternocleidomastoid muscle to the tonsillar fossa through the internal and external carotid arteries. It lacks specific symptoms and is prone to clinical misdiagnosis or missed diagnosis.^[5]^ Clinically, most children present with painless cervical masses; however, cases of snoring during sleep as the initial symptom have not been reported. In this study, we describe an abnormal case of a second BCC with snoring during sleep as the initial symptom and review the literature.

**Figure 1:**
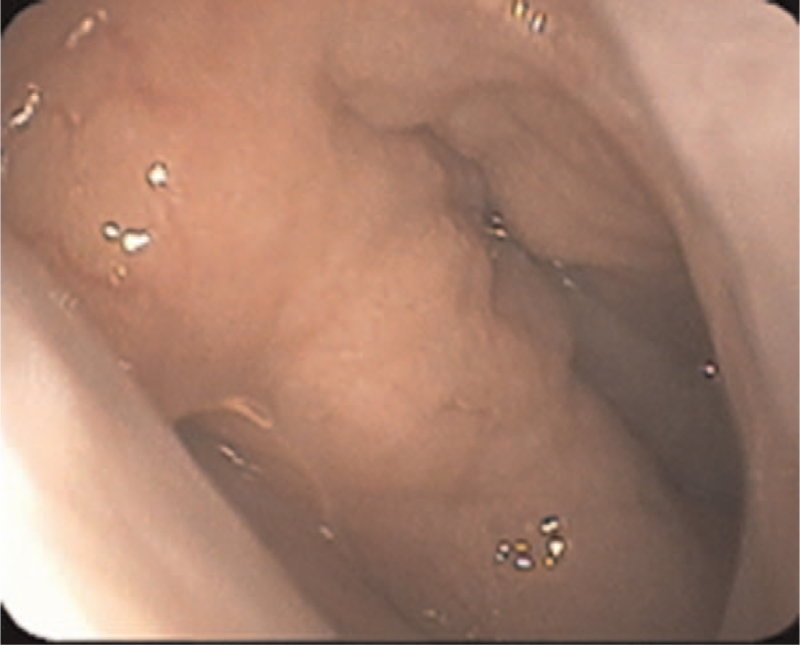
Preoperative electronic laryngoscopy shows that the right pharyngotympanic tube is protruded at the round pillow.

**Figure 2:**
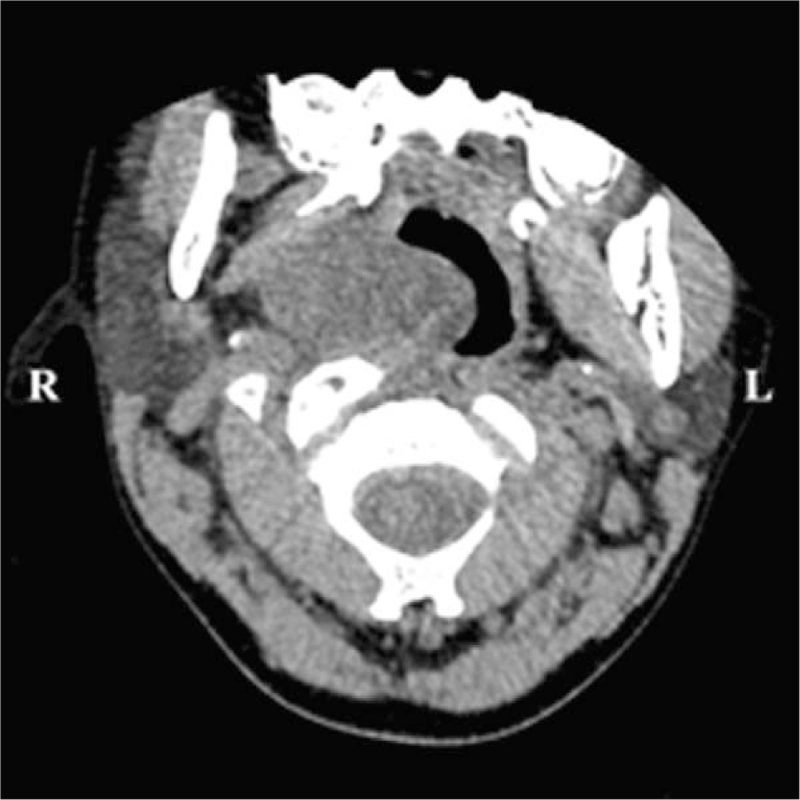
Preoperative plain CT scanning of the neck presents an oval soft tissue density shadow in the right pharyngeal wall, with CT value of about 31 HU.

**Figure 3:**
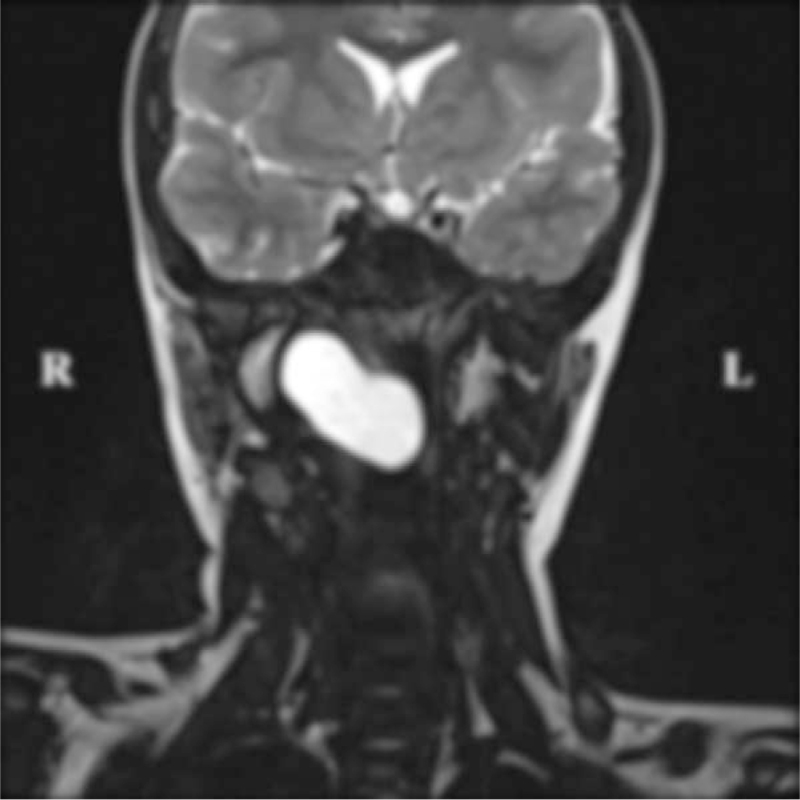
Preoperative plain and enhanced MRI of the nasopharynx display that the right parapharyngeal space extends to the nasopharyngeal cavity, there is a cystic isolow signal intensity on T1WI and high signal intensity on T2WI, and fat suppression shows high signal intensity on T2WI.

A child (male, 9 years and 5 months old) was admitted on November 13, 2019, because of snoring for more than 3 years, aggravation for 2 months, and a nasopharyngeal mass detected for 1 week. Three years before admission, the child gradually presented with nasal congestion and snoring, occasionally accompanied by mouth breathing secondary to catching cold. In the last 2 months, the above symptoms aggravated daily. One week before admission, physical examination in the outpatient department of our hospital revealed a smooth mass in the right nasopharynx, without cough, expectoration, swallowing pain, foreign body sensation, bleeding, or fever. Special examination showed slightly congested pharyngeal mucosa and a mass behind the right palatopharyngeal arch, with a diameter of approximately 3 cm, smooth surface, and no rupture. Electronic laryngoscopy (Fig. 1) suggested rhinitis and a pharyngeal mass. Cervical CT (Fig. 2) revealed an oval soft tissue density shadow in the right pharyngeal wall, with even density, unclear boundaries with the soft tissue of the lateral pharyngeal wall, a size of approximately 21 × 41 × 30 mm, and partially protruding to the nasopharynx and oropharynx. Plain and enhanced magnetic resonance imaging (MRI) of the nasopharynx (Fig. 3) showed that the right parapharyngeal space extended to the nasopharyngeal cavity, there was a cystic low signal intensity on T1WI and high signal intensity on T2WI, and fat suppression showed high signal intensity on T2WI, with uniform signals, clear boundaries, and a size of approximately 18 × 38 × 30 mm, which was considered as BCC (type II). After admission, no abnormalities were found during the routine examinations. A nasopharyngeal mass (right) was clinically diagnosed, and a second BCC was suspected.

**Figure 4:**
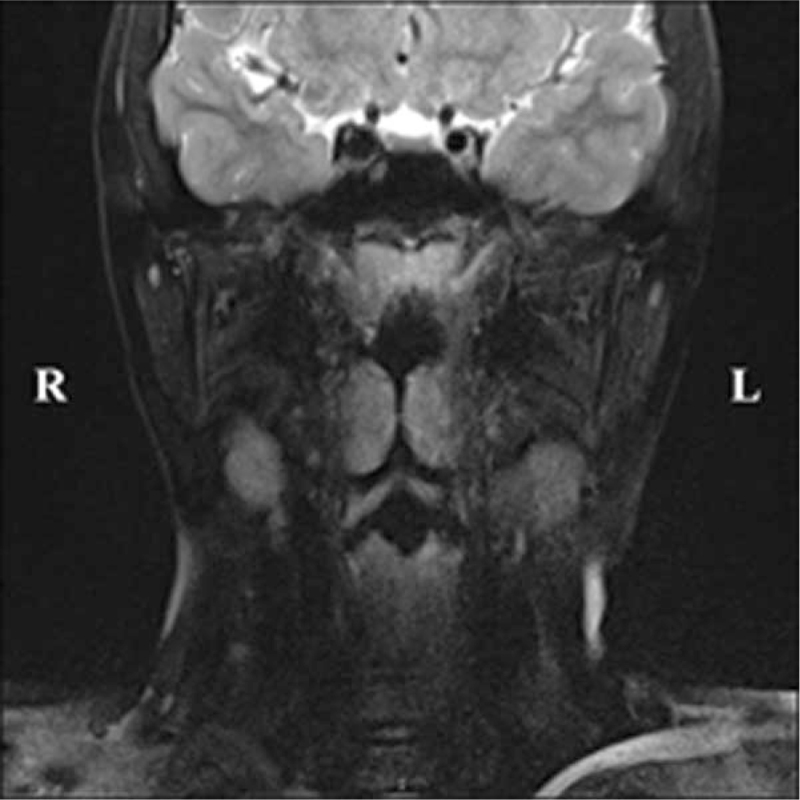
Plain MRI of the nasopharynx 6 months after the surgery demonstrates that the original cystic mass is not found. MRI = magnetic resonance imaging.

On November 19, 2019, the child underwent endoscopic BCC resection under general anesthesia. During surgery, the soft palate was suspended to expose the mass fully. The lower end of the mass was parallel to the palatopharyngeal arch, and the right pharyngotympanic tube protruded at the round pillow. After fully exposing the mass, the normal mucosa around the cyst was incised with an electric knife, the bleeding point was fully stopped by bipolar electrocoagulation, and the mass was separated along the cystic wall using a detacher under the endoscope. When separating the depth, the cyst ruptured and the cystic fluid flowed out, and separation continued along the cystic wall to the cystic root, followed by cutting off. Postoperative pathology confirmed that the right pharyngeal mass was the second most common BCC. Six months after the surgery, nasopharyngeal MRI (Fig. 4) demonstrated that the original cystic mass was not found after surgery for the right BCC. Postoperatively, the child was followed up for 1 year, and snoring during sleep improved significantly, without pharyngeal discomfort or recurrence.

This case report was approved by the review committee of Shenzhen Children's Hospital, and informed consent was obtained from the patients’ parents.

## 2 Discussion

The second BCC was first reported by Bailey in 1929 and is a residue formed by incomplete degeneration of the second branchial arch. Because cysts take a long time to expand and appear in the clinic, they always appear later, and their diagnosis is difficult.^[5–7]^ The second BCC can occur at any age, without obvious gender tendencies. It usually occurs in children, mostly in the neck. Its growth can compress important blood vessels or tracheae in the neck, causing symptoms that lack specificity.^[6]^ A typical second BCC is presented as a cervical mass in the anterior one-third of the sternocleidomastoid muscle and may have an open passage to the pharynx. Based on the relationship between the second BCC and the surrounding tissues of the neck, Bailey classified it into the following 4 types: Type I cysts are located in the deep surface of the platysma and the anterior margin of the sternocleidomastoid muscle; type II cyst is located in the deep surface of the sternocleidomastoid muscle, lateral carotid space, and behind the mandibular gland; type III cyst is located between the internal carotid and the external carotid; type IV cyst is close to the pharyngeal mucosal wall and can extend upward to the skull base.^[7]^ Among them, type I was the most common, while type IV was rare. In this case, the initial symptoms of the child were snoring during sleep. The pharyngeal tumor originated from the right parapharynx and had a smooth surface. According to the classification, it was classified as rare type IV and second BCC.

The second BCC lacks specific clinical or imaging features and can only be definitively diagnosed by surgical resection and biopsy. Preoperative ultrasound examination of the neck is simple, convenient, and rapid, but it is not good to display the details of the anatomical structure around the cyst. It has been reported that the sensitivity, specificity and accuracy of color Doppler ultrasonography in detecting BCCs are 91.67%, 50% and 90%, respectively.^[8]^ Imaging examination also has good application value in the diagnosis of second BCC and can make localization and qualitative diagnosis. Typical CT findings of the patients show a homogeneous low-density cystic mass with clear boundaries and a uniform surrounding thin and smooth wall. MRI findings present that cystic fluid show low to slightly high signal intensity on T1WI and high signal intensity on T2WI.^[9,10]^

At present, the second BCC is mainly treated by complete surgical resection at home and abroad.^[6]^ Surgical methods mainly include traditional surgery and minimally invasive surgery. Although traditional surgery can fully expose the mass through full-thickness incision and separation in the neck, there is a large amount of intraoperative blood loss and a large scar after surgery. Minimally invasive surgery is directly performed in the neck through a small incision approach without cutting off the muscles, which reduces surgical trauma and completely removes the cyst by reducing the cyst. However, endoscopic BCC resection has not been reported in the literature. In this case, the visible cyst and cystic wall were completely resected under the endoscope, with intraoperative blood loss of approximately 10 mL, no loss of peripheral blood vessels or nerves, and good postoperative prognosis. No recurrence occurred during the follow-up.

The second BCC lacks typical symptoms, and its clinical manifestations are complex and diverse. Among them, the second BCC with snoring during sleep as the initial symptom is relatively rare. Lacking relevant knowledge, otolaryngologists are prone to missed diagnosis or misdiagnosis. In this study, a case of a second BCC with snoring during sleep was reported as the main manifestation. Endoscopic BCC resection was performed, and the postoperative efficacy was good. However, more cases need to be considered.

## Author contributions

**Conceptualization:** Zhixiong Xian, Saihong Han, Lan Li.

**Data curation:** Zhixiong Xian, Yongchao Chen.

**Formal analysis:** Yishu Teng, Lan Li.

**Investigation:** Yongchao Chen.

**Project administration:** Yishu Teng.

**Resources:** Yishu Teng.

**Supervision:** Yongchao Chen, Zhixiong Xian.

**Visualization:** Saihong Han, Lan Li.

**Writing – original draft:** Zhixiong Xian, Yongchao Chen, Yishu Teng, Saihong Han.

**Writing – review & editing:** Yishu Teng, Saihong Han.
